# Identification of a novel CNV at the *APC* gene in a Chinese family with familial adenomatous polyposis

**DOI:** 10.3389/fmolb.2023.1234296

**Published:** 2023-07-27

**Authors:** Juyi Li, Chengzhi He, Jing Gong, Xiufang Wang, Chao Liu, Aiping Deng, Lin Zhu

**Affiliations:** ^1^ Department of Pharmacy, The Central Hospital of Wuhan, Tongji Medical College, Huazhong University of Science and Technology, Wuhan, Hubei, China; ^2^ Department of Gastrointestinal Surgery, The Central Hospital of Wuhan, Tongji Medical College, Huazhong University of Science and Technology, Wuhan, China; ^3^ Department of Gastroenterology, The Central Hospital of Wuhan, Tongji Medical College, Huazhong University of Science and Technology, Wuhan, Hubei, China; ^4^ Department of Pain, The Central Hospital of Wuhan, Tongji Medical College, Huazhong University of Science and Technology, Wuhan, Hubei, China; ^5^ Hubei Key Laboratory of Diabetes and Angiopathy, Hubei University of Science and Technology, Xianning, Hubei, China; ^6^ Department of Pediatrics, Tongji Hospital, Huazhong University of Science and Technology, Wuhan, Hubei, China

**Keywords:** familial adenomatous polyposis, adenomatous polyposis coli gene, whole-exome sequencing, copy number variations, genetic counseling

## Abstract

**Introduction:** Familial adenomatous polyposis (FAP) is the second most commonly inherited colorectal cancer (CRC) predisposition caused by germline mutations within the *adenomatous polyposis coli* (*APC*) gene. The molecular defects and clinical manifestations of two FAP families were analyzed, and individual prevention strategies suitable for mutation carriers in different families were proposed.

**Methods and results**: The pathogenic gene mutations were identified among the two families using whole-exome sequencing and verified with Sanger sequencing or quantitative polymerase chain reaction (qPCR). One novel (GRCh37:Chr5: 112145676–112174368, del, 28,692 bp) and a known (c.C847T:p.R283X) mutation in the *APC* gene were pathogenic mutations for FAP, according to the sequencing data and tumorigenesis pattern among the family members. The two mutations led to a premature translational stop signal, synthesizing an absent or disrupted protein product.

**Conclusion**: Our findings expand the known germline mutation spectrum of the *APC* gene among the Chinese population. This reaffirms the importance of genetic testing in FAP. Genetic consultation and regular follow-ups are necessary for the individualized treatment of cancer-afflicted families with APC expression deficiency. Additional work is required to develop safe and effective chemotherapy and immunotherapy for FAP based on the mutation type.

## 1 Introduction

Familial adenomatous polyposis (FAP) is a hereditary colorectal disease subtype with a poor prognosis. Colorectal cancer (CRC) predisposition syndrome is rare, characterized by 100 s–1,000 s of adenomas developing in the colon and rectum with their onset in childhood and adolescence. Moreover, CRC possesses associated extracolonic manifestations, such as desmoid tumors, dental and skin abnormalities, retinal spots, and malignant tumors from other organs (Carr and Kasi, 2022). Surgery is an effective therapy for FAP patients with colonic disorders, and regular chemotherapy has been shown to benefit FAP patients with the postoperative pathological diagnosis of adenocarcinoma (Tougeron et al., 2020). Recent colorectal cancer guidelines indicate that the type of pathogenic gene mutation is related to the patient’s prognosis and responses to chemotherapy and immunotherapy (Stjepanovic et al., 2019).

FAP follows an autosomal dominant inheritance pattern caused by the monoallelic mutation in the *adenomatous polyposis coli* (*APC*) gene (Recio-Boiles and Cagir, 2022). APC is a tumor suppressor gene located on chromosome 5q21, encoding a large scaffolding protein with functions in cell cycle regulation, apoptosis, transcription, and cell migration (Kim and Bodmer, 2021). *APC* comprises 16 exons; the last exon encodes nearly 70% of the APC protein. FAP frequency is approximately 1:8,000 in the general population with almost complete penetrance, affecting multiple family generations (Bisgaard et al., 1994).

Currently, more than 2,000 pathogenic variations in the *APC* gene are found, the majority of which are observed in the 5′-ends of exon 15, also called the mutation cluster region (MCR) (Schirosi et al., 2013; Jung et al., 2016), and are situated between 1286 and 1513 codons (Cetta and Dhamo, 2007). Codons 1309 and 1061 are hotspots for mutation, accounting for nearly 17% and 11% of all germline *APC* mutations, respectively (Leoz et al., 2015). Compared to mutation patients outside the MCR, those with MCR mutations typically have a worse prognosis, manifesting their condition early (Ghadamyari et al., 2021). In a family, identifying the specific *APC* mutation can help targeted sequencing testing for presymptomatic at-risk family members (Cruz-Correa et al., 2017).

Identifying patients and families at an extremely high risk of developing cancer can help reduce cancer occurrence and mortality. Several studies described an association between *APC* mutation localization and the phenotype among FAP patients (Heinen, 2010). The diagnosis and patient follow-up could be improved by connecting the genotypes to the phenotypes. However, there is a paucity of information on the genotypic spectrum and clinical characterization of FAP in China. One novel and one known *APC* mutation were reported in two Chinese families with FAP. Our study aimed to analyze the molecular defects and clinical manifestations in the two families, for appropriate personalized prevention strategies against all mutation carriers.

## 2 Results

### 2.1 Clinical characteristics

**FIGURE 1:**
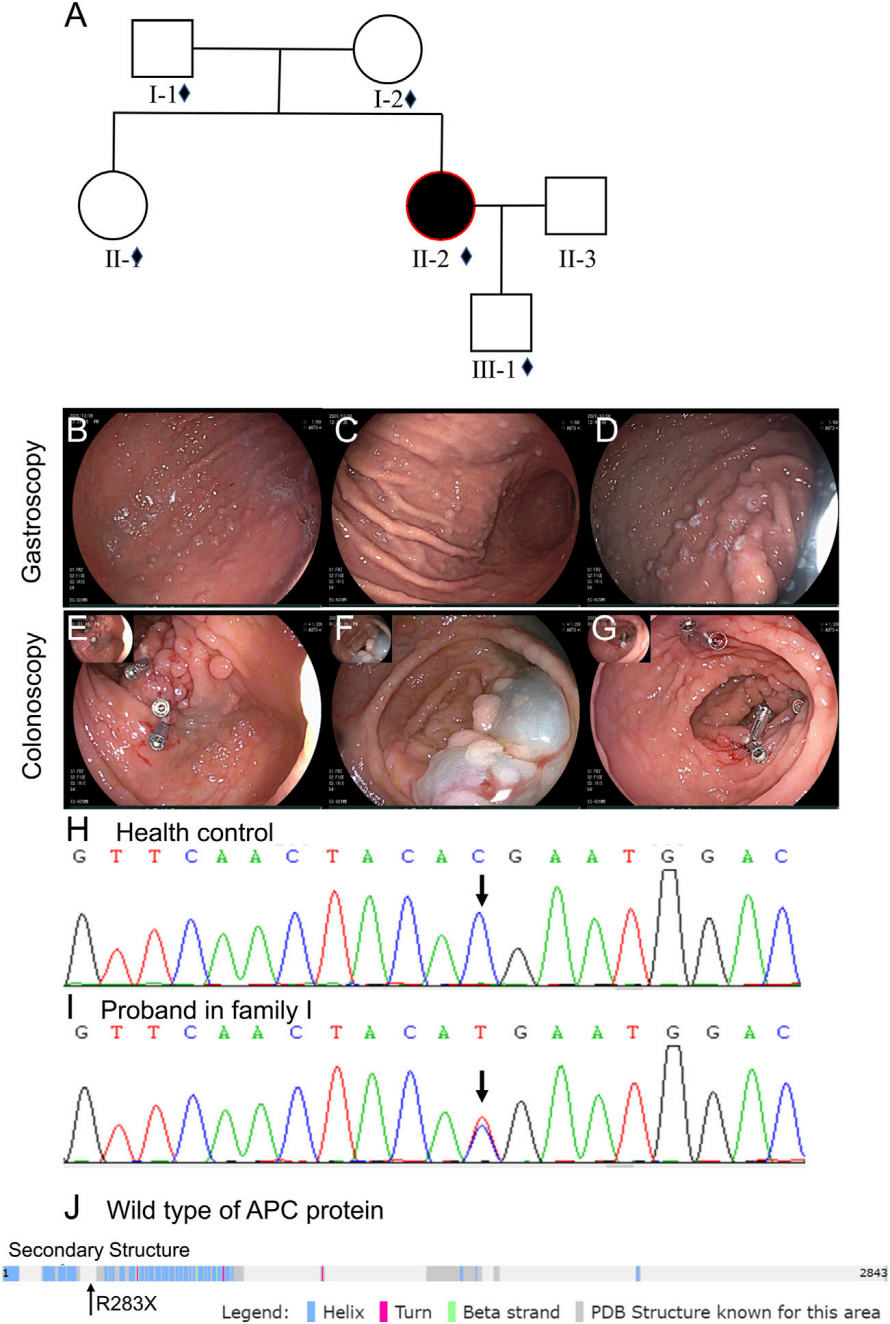
Clinical data of family I. **(A)** Pedigree structure of family I: the red ring depicts the proband, squares indicate male members, and circles represent female members. Black shading represents FAP individuals. The small diamonds denote the relatives whose DNA was available for testing. **(B–D)** Gastroscopy of the proband. **(E–G)** Colonoscopy of the proband. **(H,I)** Sanger sequencing analysis of the proband’s *APC* gene (c.C847T:p.R283X): the C base at position 847 of the *APC* gene is replaced by the T base. **(J)** Location of the p.R283X mutation in the secondary structures of the APC protein.

The detailed pedigree of family I is given in Figure 1A. The proband was a 34-year-old female, whose first symptom of altered bowel movement appeared at 31 years (July 2019). She underwent her first gastrointestinal endoscopy and treatment that year. This involved the endoscopic dissection of colonic polyps and gastric polypectomy. Biopsy indicated multiple tubular adenomas inside the ascending colon and rectum. The patient underwent three more gastrointestinal endoscopy examinations and treatment in the following 2 years. The last endoscopy examination (6 December 2021, Figure 1) showed multiple glandular polyps inside the gastric body and fundus, with tubular adenoma and adenomatous polyp inside the ascending colon and tubular adenoma in the transverse colon with mild-to-moderate heteroplasia of the focal glandular epithelium. However, no extracolonic manifestations appeared, such as desmoid tumors or dental and skin abnormalities. The possibility of classic FAP was highly suspected with her clinical manifestations, so whole-exome sequencing was conducted.

**FIGURE 2:**
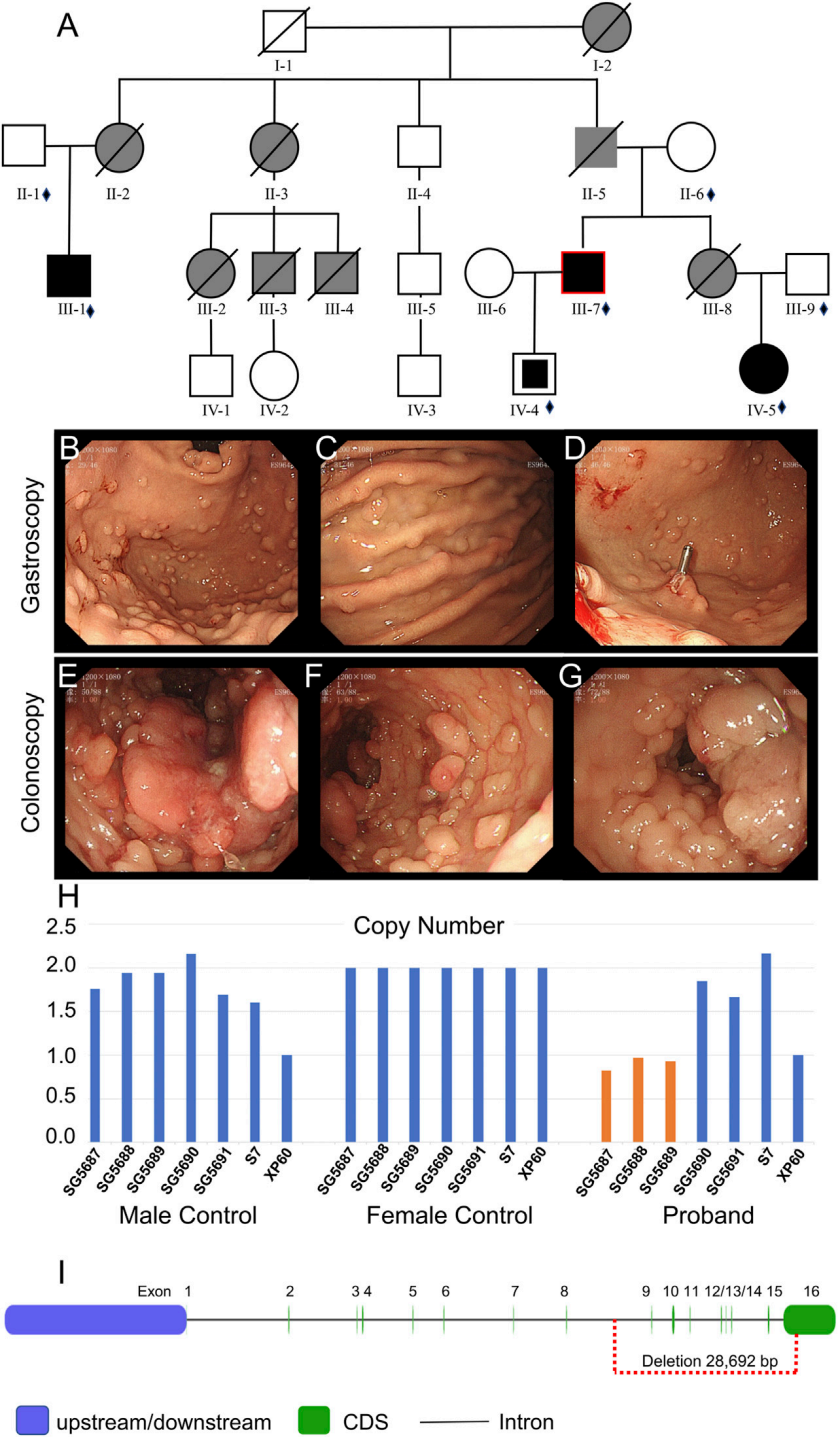
Clinical data of family II. **(A)** Pedigree structure of family II: the red ring indicates the proband, squares indicate male members, circles indicate female members, and crosses indicate deceased individuals. Dark shading represents individuals with FAP, partly dark shading represents mutation carriers, and gray shading represents CRC without the gene test. The small diamonds denote the relatives whose DNA was available for testing. **(B–D)** Gastroscopy of the proband. **(E–G)** Colonoscopy of the proband. **(H)** qPCR of the proband’s peripheral blood DNA to verify the CNV in *APC* (GRCh37:Chr5: 112145676–112174368, del, 28,692 bp). **(I)** Location of the GRCh37:Chr5: 112145676–112174368 delete mutation in the *APC* gene.

Family II is a typical and interesting large family. The detailed pedigree is given in Figure 2A, and the clinical characteristics of the family members are listed in Supplementary Table S2. The proband of family II sought medical attention for bloody stools at 41 years (July 2019). Gastrointestinal endoscopy showed multiple new organisms in the stomach and colon (Figure 2), and biopsy suggested adenocarcinoma of the transverse colon. The glandular epithelium of the mucosa showed moderate-to-severe dysplasia of approximately 40 cm from the anal margin. A fragmentary villous or serrated glandular epithelium was observed with low-grade intraepithelial neoplasia of approximately 10 cm from the anal margin. In August 2019, laparoscopic total colectomy and ileostomy were conducted. The pathological examination suggested the following: 1. multiple neoplasm and ulcerated areas were carcinomatous, with medium well-differentiated adenocarcinoma. The deepest cancer infiltration reached the outer membrane (the largest mass was 4 × 3.5 cm), with tumor plugs in the vascular area. 2. There were no cancer cells in the colon tissues of the two broken ends, and the local glandular epithelium of the broken ends indicated low-grade intraepithelial neoplasia. 3. Metastatic cancer was found in 3/14 of the peri-intestinal lymph nodes. Oxaliplatin chemotherapy was performed several times after the operation. Over the next year or so, multiple gastroscopies and colonoscopies were performed.

**FIGURE 3:**
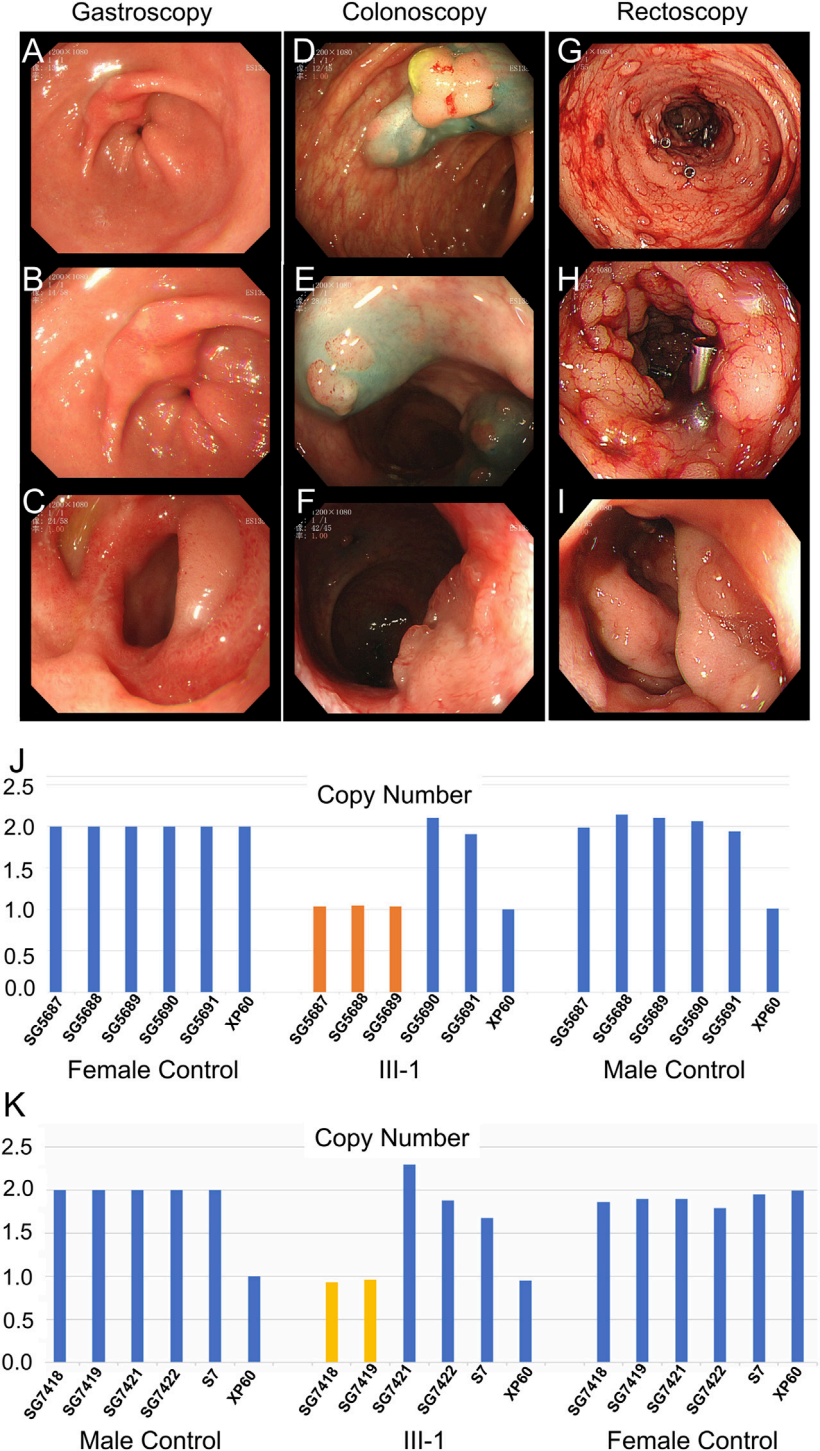
Clinical data of III-1 (the proband’s brother) of family II. **(A–C)** Gastroscopy of III-1. **(D–F)** Colonoscopy of III-1. **(G–I)** Rectoscopy of III-1. **(J)** qPCR of III-1’s peripheral blood DNA to verify the CNV in *APC* (GRCh37:Chr5: 112145676–112174368, del, 28,692 bp). **(K)** qPCR of III-1’s DNA extracted from newly developed polyps to verify the CNV in *APC* (GRCh37:Chr5: 112145676–112174368, del, 28,692 bp).

The father (II-5), aunts (II-2 and II-3), sister (III-8), and cousins (III-2 and III-3) of the proband were all diagnosed with colon or rectal cancer and were deceased. The brother of the proband (III-1) was hospitalized at 32 years (December 2020) due to irregular stools, diarrhea, and stool blood. Five gastrointestinal endoscopy examinations and treatments were performed, among which the gastrointestinal endoscopy examination performed in December 2020 is depicted in Figure 3. Gastroscopy (Figures 3A–C) indicated duodenal bulb ulcer (stage A2) and erosive gastritis (grade 2). Biopsy suggested severe chronic inflammation of the gastric antrum mucosa accompanied by erosive gastritis with mild activity. Colonoscopy (Figure 3D–F) revealed multiple neoplasms of the large intestine. EMR and argon coagulation were performed. Biopsy of the ascending colon revealed adenomatous polyps with multiple adenomatous polyps at the rectum and sigmoid colon junction. Rectal eminence was observed under a rectoscope (Figures 3G–I), and a biopsy revealed low-grade intraepithelial neoplasia of superficial mucosal glands with polyps inside the anal canal.

**FIGURE 4:**
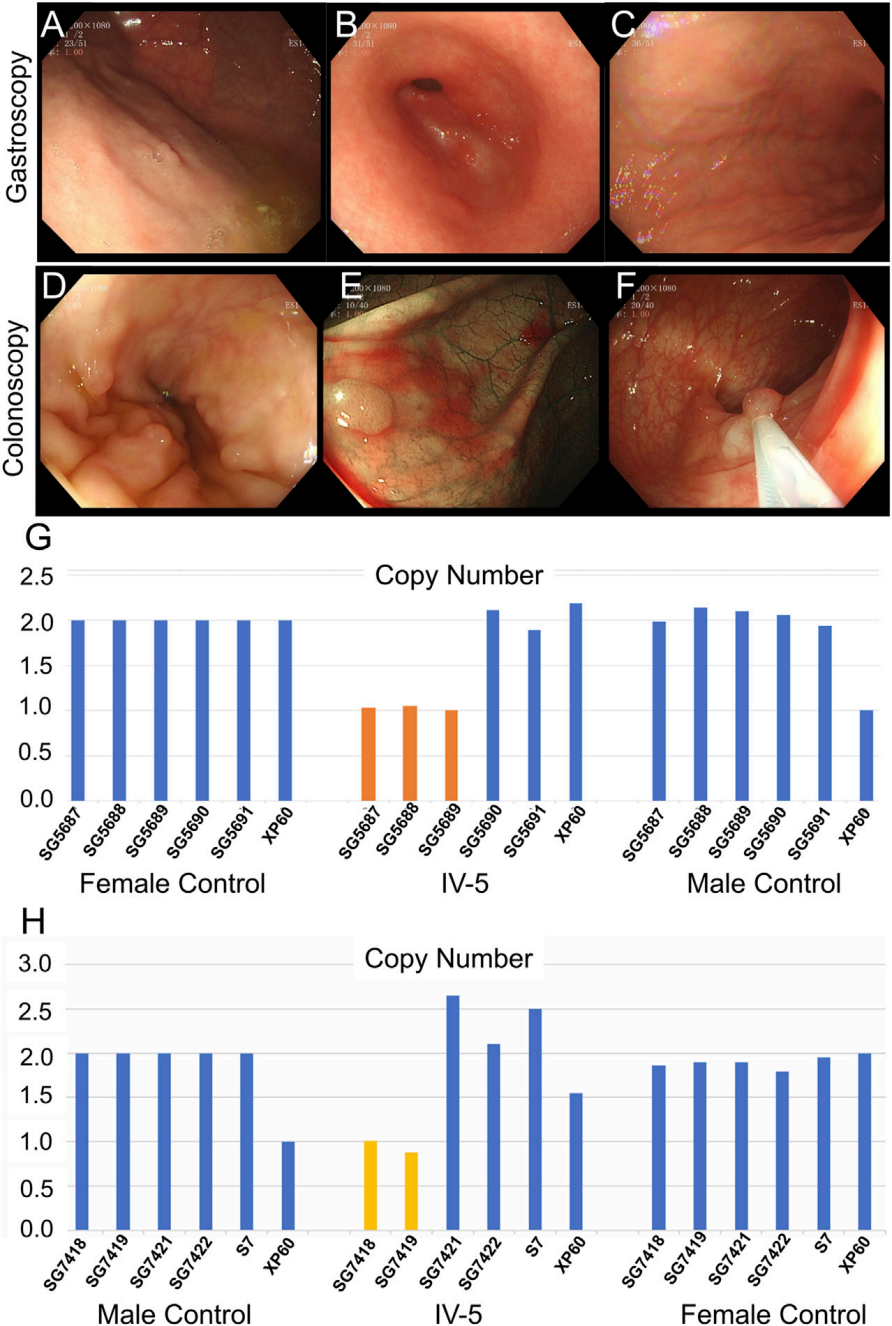
Clinical data of IV-5 (the proband’s niece) in family II. **(A–C)** Gastroscopy of IV-5. **(D–F)** Colonoscopy of IV-5. **(G)** qPCR of IV-5’s peripheral blood DNA to verify the CNV in *APC* (GRCh37:Chr5: 112145676–112174368, del, 28,692 bp). **(H)** qPCR of IV-5’s DNA extracted from newly developed polyps to verify the CNV in *APC* (GRCh37:Chr5: 112145676–112174368, del, 28,692 bp).

The 22-year-old niece (IV-5) of the proband underwent gastrointestinal polyp extraction at 17 years. After surgery, gastrointestinal endoscopy was performed several times. Gastroscopy indicated multiple gastric polyps treated with argon coagulation and erosive gastritis (grade 1) (Figures 4A–C). Biopsy depicted mild-to-moderate chronic inflammation of the gastric antrum mucosa showing mild activity. A colonoscopy (March 2021) showed multiple large intestine polyps, and EMR and argon coagulation were conducted (Figures 4D–F). Biopsy revealed adenomatous polyps inside the transverse colon. The 6-year-old son (IV-4) of the proband is currently asymptomatic; he is too young to develop clinical symptoms. Whole-exome sequencing was performed for proband II and quantitative polymerase chain reaction (qPCR) for the other family members associated with the disease characteristics of family II members.

### 2.2 Genetic test results

The whole-exome sequencing data of the proband in family I are described in Supplementary Table S3A. Approximately 117,621 mutations were identified with 106,580 SNPs and 11,041 indels. The number of synonymous and missense mutations, new SNPs, and indels was 11,015, 9,886, 628, and 676, respectively. We identified a known mutation NM_000038:exon9:c.C847T:p.R283X of *APC* (rs786201856; accession: VCV000836957.3) (Hong et al., 2021a; Hong et al., 2021b). In this mutation, the C base at position 847 of the *APC* gene was replaced by the T base (Figure 1H/I). This led to the premature emergence of a stop codon, premature APC protein translation termination, and protein function loss (Figure 1J), showing the specific clinical significance of this mutation.

Sanger sequencing helped detect the APC mutation (NM_000038:exon9:c.C847T:p.R283X) in the proband and family members of the patient to provide genetic counseling. The germline mutation in *APC* was not observed in other family members (I-1, I-2, II-1, and III-1). Thus, the APC mutation of the proband is spontaneous and not passed on to her son.

Whole-exome sequencing was also performed on the proband in family II (Supplementary Table S3B). Approximately 114,508 mutations with 104,007 SNPs, 10,485 indels, and 16 copy number variations (CNVs) were identified. The number of new SNPs and indels was 656 and 656, respectively. We verified the proband harboring a new CNV loss within the *APC* tumor suppressor gene (GRCh37:Chr5: 112145676–112174368, del, 28,692 bp) (Figure 2I) using qPCR (Figure 2H). The brother (III-1), niece (IV-5), and son (IV-4) of the proband possessed the same mutation (Figure 3J; Figure 4G; Supplementary Figure S1A). The mutation was detected in the first two polypous tissues (Figures 3K, 4H). In contrast, this mutation was not observed in the other family members (Supplementary Figures S1B–D). Therefore, the substantial deletion of the *APC* gene in this family is the causative mutation for polyposis. FAP is a dominant syndrome caused by truncating mutations or large deletions.

## 3 Discussion

We identified one novel mutation (GRCh37:Chr5: 112145676–112174368, del) and a known mutation (NM_000038:exon9:c.C847T:p.R283X, rs786201856) within the *APC* gene in two typical FAP families, which were pathogenic for FAP.

*APC* is a tumor suppressor gene, promoting the rapid degradation of CTNNB1 (cadherin-associated protein, beta 1). *APC* participates in Wnt signaling as a negative regulator. Currently, more than 10,000 *APC* mutations have been identified. There are several important observations regarding *APC* mutations: 1) the vast majority of mutations discovered would lead to *APC* product truncation; 2) most mutations occurred in the first half of the coding sequence, and somatic mutations in colorectal tumors were concentrated in the MCR; 3) transition of cytosine to other nucleotides was the most common point mutations in the *APC* gene; and 4) there was a correlation between the germline mutations of FAP1 patients and colorectal polyps. For most adenomas and carcinomas in the colon and rectum and some in the stomach, inactivating both alleles of the *APC* gene is necessary as an early event.

During the diagnosis of colorectal cancer in the 34-year-old female patient, our patient from family I had more than 100 adenomatous colorectal polyps with gastric polyps. However, there were no additional extracolonic signs linked to FAP (desmoids, osteomas, cutaneous soft-tissue tumors, dental abnormalities, and CHRPE). The *APC* mutation is denoted by *APC* c.847C>T in the proband at the cDNA level and p.Arg283Ter (R283X) at the protein level. It has been reported in sporadic and syndromic adenocarcinoma patients within the COSMIC database (Tate et al., 2019) and is considered pathogenic by ClinVar (Landrum et al., 2018). Additionally, this mutation has been clarified in FAP patients as having adamantinomatous craniopharyngiomas.

The c.847C>T pathogenic mutation, also called p.R283X, affects the coding exon 8 of the *APC* gene and is caused by a C-to-T substitution at the nucleotide position 847. This mutation converts the amino acid within coding exon 8 from an arginine to a stop codon. This harmful variation exists in many families across several ethnic groups with familial adenomatous polyposis (Owen et al., 2018; de Oliveira et al., 2019). Other than the clinical information in the literature, this change is anticipated to cause function loss by nonsense-mediated mRNA decay (NMD) or premature protein truncation. This variant has been reported in 15 probands, meeting eight phenotype points, and in 50 probands with FAP not otherwise specified, meeting more than 16 phenotype points in total. It has been reported to segregate with FAP in five meioses from one family and in 31 members from one large FAP family (Mohamed et al., 2003). In summary, this variant meets the criteria to be classified as pathogenic for FAP based on the ACMG/AMP criteria applied. The mutation of the proband was not passed on to her son, and she is expecting her second child. However, the second child probably carried the disease-causing mutation. Genetic counseling was recommended according to her genetic mutation.

Family II is a typical FAP family. The genomic region of the *APC* gene is deleted out-of-frame in family II (GRCh37:Chr5: 112145676–112174368, del, 28,692 bp). The consequence could be a missing or disrupted protein product with a premature translational stop signal. This variation has not been documented among people with *APC*-related diseases. Pathogenic loss-of-function mutations in *APC* exist (Lagarde et al., 2010), which are labeled pathogenic based on these factors. The elder brother (III-1) and niece (IV-5) of the proband were mutant gene carriers; both were of marriageable age, but neither was married. They were concerned that their future children would carry the same gene mutation. Therefore, according to the principle of eugenics, genetic counseling is necessary, for the third generation of test-tube babies before pregnancy.

Nearly all FAP patients develop CRC if not identified and treated early (Half et al., 2009). Adjuvant chemotherapy and immunotherapy are required in most instances of FAP after total colectomy, similar to sporadic colorectal cancer and Lynch syndrome (Zhang et al., 2019; Koskenvuo et al., 2020; Kemp Bohan et al., 2021; Rohani et al., 2022). Cancer research significantly emphasizes the therapeutic targeting of abnormal beta-catenin activity. Creating medicines that target cancer cells, while having acceptable safety profiles, has remained a pharmacological challenge since Wnt signaling is a highly conserved system in normal cellular physiology. Early clinical trials of several medicines that inhibit upstream effectors in the Wnt signaling pathway have been undertaken, albeit with worries about off-target effects. In the phase 1 clinical trial of paclitaxel and vantictumab, a monoclonal antibody against Fzd receptors, was used to suppress Wnt signaling by preventing binding with all Wnt ligands. The trial indicated moderate efficacy among Wnt-upregulated metastatic breast (Diamond et al., 2020) and pancreatic (Davis et al., 2020) cancer. However, concerns have been raised about the safety of the drug concerning the bones. Furthermore, porcupine inhibitors, an enzyme that processes Wnt signaling proteins, have been created to combat Wnt-driven cancers. Most notably, WNT974 has demonstrated safety but limited efficacy in advanced solid tumors (Rodon et al., 2021). However, the effectiveness of focusing on upstream targets is still debatable due to tumor resistance with more downstream alterations, including CTNNB1- or *APC*-mutated malignancies. There are serious concerns about off-target consequences, such as damage to normal intestinal tissues and directly inhibiting beta-catenin or *APC* (Kahn, 2014; Zhang et al., 2016; Lai and Kahn, 2021; Wang et al., 2021). Hence, much research on cancer is required to create safe and efficient Wnt signaling inhibitors.

Our study contributes to identifying FAP genotypes in China, with significant implications for genetic counseling, diagnosis, cancer prevention, and treatment. Whole-exome sequencing is a rapid, accurate, and reliable technique to identify genetic variants in suspected FAP patients. It has various potential applications in the genetic testing of FAP-related tumors. Thus, abnormal Wnt signaling, including *APC* mutations, can be a promising target for the development of chemotherapy and immunotherapy against FAP.

## 4 Materials and methods

### 4.1 Patients

The Ethics Committee of the Central Hospital of Wuhan approved the study, with the ethical approval code of 2020-192. All subjects included in this analysis were informed in person, and their written informed consent was obtained. The two probands, diagnosed with FAP, were recruited from the Department of Gastrointestinal Surgery at the Central Hospital of Wuhan. The clinical diagnosis of FAP was confirmed by a gastroenterologist using multiple gastroscopic and colonoscopic biopsy reports, clinical investigations, and a detailed family pedigree.

### 4.2 Mutation analysis

We extracted the genomic DNA from the peripheral blood or newly developed polyps of each proband and part of the family members. DNA fragments were sequenced using a high-throughput sequencer (Illumina HiSeq 2500 Analyzer; Illumina, CA, United States). As previously demonstrated, single-nucleotide variant, insertion, and deletion queries were performed (Li et al., 2017). The reference genome for whole-exome sequencing was UCSC hg19, NCBI build 37. PCR amplification and Sanger sequencing confirmed the detected variants, which helped in segregation analysis.

Samples were detected using next-generation sequencing for suspected CNVs. The quantitative polymerase chain reaction system using Roche LightCycler^®^ II 480 Probes Master mix, as per the manufacturer’s instructions, was applied to verify the result accuracy and determine the breakpoint positions. Primer sequences used are listed in Supplementary Table S1.

## Data Availability

The datasets presented in this study can be found in online repositories. The names of the repository/repositories and accession number(s) can be found in the article/Supplementary Material.
