# Supplementary material for: Identification of a novel CNV at the APC gene in a Chinese family with familial adenomatous polyposis
Source: Front Mol Biosci. 2023 Jul 27;10:1234296. doi: 10.3389/fmolb.2023.1234296 (PMC10415011; doi:10.3389/fmolb.2023.1234296)
Supplement: Supplementary file 1 [file Table1.DOCX]

## Supplementary Table 1 Location in the genome and primers of amplicons

| Amplicon | Genomic location (hg19) | Gene | Exon/Intron location | Primer sequence F | Primer sequence R | Notes |
| --- | --- | --- | --- | --- | --- | --- |
| Family I |  |  |  |  |  |  |
| / | chr5：112815507 | APC | Exon8 | ATCAATGAACTTATCTGAATGTGG | CATTAGTGACCAGGGTTTGT | / |
| Family II |  |  |  |  |  |  |
| SG5690 | chr5：82807979-82808060 | VCAN | Exon6 | AGTGTGAAAACCAGGATGCC | TAATCGCACTGGTCAAAGCC | Upstream of deletion |
| SG5687 | chr5：112154717-112154860 | APC | Exon10 | ATGTCGCGAACTTTGCTAGC | TTTACTGCCCCGGGAATTTC | Deletion region  chr5:112145676-12174368 |
| SG5688 | chr5：112170725-112170844 | APC | Exon15 | TGCTGTAGATGGTGCACTTG | GCTATCAAGCTGGACACATTCC |  |
| SG5689 | chr5：112162813-112162909 | APC | Exon12 | CAGGCCATTGCAGAATTATTGC | AAGCCATTCCAGCATATCGTC |  |
| SG5691 | chr5：138857945-138858062 | TMEM173 | Exon6 | GCATTACAACAACCTGCTACGG | ATCCAGGAAGCGAATGTTGG | Downstream of deletion |
| XP60 | chrX：43809165-43809335 | NDP | / | GTGACAGGAGGAACGGAAGGGTT | CTGTTTTACCTGGCTAAGGTTGTG | X chromosome control |
| S7 | chr7：5568571-5568704 | ACTB | / | GCCGTTTTCCGTAGGACTCT | CCCACAACACTGTCTTAGACACC | Autosomal control |
| GAPDH | chr12：6643539-6643702 | GAPDH | / | TACTAGCGGTTTTACGGGCG | GAACAGGAGGAGCAGAGAGCG | Autosomal control |

Verification of the mutation in family I and copy number variation in family II: Three amplicon were designed in the deletion region of *APC* gene, one control amplicon was designed in the upstream and downstream of the deletion region of *APC* gene, and one control amplicon was designed on X chromosome. Relative DNA quantization of the above and control amplicon was carried out by q-PCR.
