# Supplementary material for: Identification of a novel CNV at the APC gene in a Chinese family with familial adenomatous polyposis
Source: Front Mol Biosci. 2023 Jul 27;10:1234296. doi: 10.3389/fmolb.2023.1234296 (PMC10415011; doi:10.3389/fmolb.2023.1234296)
Supplement: Supplementary file 3 [file Table2.DOCX]

## Supplementary Table 2 Clinical characteristics of part members in family II

|  | Gender | FAP diagnosis | First symptoms | Surgery | FAP phenotype | Germline mutation |
| --- | --- | --- | --- | --- | --- | --- |
| Proband  (III-7) | M | 42 years old | Bloody stool | Yes | Classic | Chr5: 112145676-112174368, del |
| uncle-in-law (II-1) | M | - | Asymptomatic | No | - | None |
| Mother  (II-6) | F | - | Asymptomatic | No | - | None |
| Cousin  (III-1) | M | 33 years old | Bloody stool | Yes | Classic | Chr5: 112145676-112174368, del |
| Brother-in-law  (III-9) | M | - | Asymptomatic | No | - | None |
| Son  (IV-4) | M | - | Asymptomatic | No | - | Chr5: 112145676-112174368, del |
| Niece  (IV-5) | F | 18 years old | Bloody stool | Yes | Attenuated | Chr5: 112145676-112174368, del |
