# Supplementary material for: Identification of a novel CNV at the APC gene in a Chinese family with familial adenomatous polyposis
Source: Front Mol Biosci. 2023 Jul 27;10:1234296. doi: 10.3389/fmolb.2023.1234296 (PMC10415011; doi:10.3389/fmolb.2023.1234296)
Supplement: Supplementary file 4 [file Table3.DOCX]

## Supplementary Table 3

## Whole-exome sequencing detail of the proband in family I

| Exome capture statistics | Proband I |
| --- | --- |
| Total (bp) | 63,757,018 (100.00 %) |
| Duplicate (bp) | 15,919,300 (24.97%) |
| Mapped (bp) | 63,675,837 (99.87%) |
| Properly mapped (bp) | 63,135,072 (99.02%) |
| PE mapped (bp) | 63,605,488 (99.76%) |
| SE mapped (bp) | 60,456,963 |
| Initial bases on target (bp) | 75,840,481 |
| Initial bases on or near target (bp) | 136,297,444 |
| Total effective yield (Mb) | 9,483.41 |
| Effective sequences on target (Mb) | 6,151.10 |
| Effective sequences near target (Mb) | 1,835.01 |
| Effective sequences on or near target (Mb) | 7,986.12 |
| Fraction of effective bases on target | 65% |
| Fraction of effective bases on or near target | 84% |
| Average sequencing depth on target | 102 |
| Average sequencing depth near target | 24.20 |
| Mismatch rate in target region | 0% |
| Mismatch rate in all effective sequence | 1% |
| Base covered on target | 60,129,788 |
| Coverage of target region | 99% |
| Fraction of target covered with at least 10x | 97% |
| Fraction of target covered with at least 50x | 76% |
| Fraction of target covered with at least 100x | 43% |
| Fraction of flanking region covered with at least 10x | 58% |
| Fraction of flanking region covered with at least 50x | 14% |
| Fraction of flanking region covered with at least 100x | 3% |
| Gender | Female |

## Whole-exome sequencing detail of the proband in family II

| Exome capture statistics | Proband II |
| --- | --- |
| Total | 68,407,844 (100%) |
| Duplicate | 14,626,705 (21.38%) |
| Mapped | 68,347,293 (99.91%) |
| Properly mapped | 67,819,858 (99.14%) |
| PE mapped | 68,297,872 (99.84%) |
| SE mapped | 98,842 (0.14%) |
| With mate mapped to a different chr | 308,928 (0.45%) |
| With mate mapped to a different chr ((mapQ>=5)) | 280,376 (0.41%) |
| Initial bases on target | 60,456,963 |
| Initial bases near target | 75,840,481 |
| Initial bases on or near target | 136,297,444 |
| Total effective yield (Mb) | 10,218.35 |
| Effective sequences on target (Mb) | 7,558.71 |
| Effective sequences near target (Mb) | 2,101.25 |
| Effective sequences on or near target (Mb) | 9,659.96 |
| Fraction of effective bases on target | 74% |
| Fraction of effective bases on or near target | 95% |
| Average sequencing depth on target | 125 |
| Average sequencing depth near target | 27.71 |
| Mismatch rate in target region | 0% |
| Mismatch rate in all effective sequence | 0% |
| Base covered on target | 60,128,594 |
| Coverage of target region | 99% |
| Base covered near target | 69,485,436 |
| Coverage of flanking region | 92% |
| Fraction of target covered with at least 10x | 97% |
| Gender | Male |
